# Supplementary material for: Estimating ecoacoustic activity in the Amazon rainforest through Information Theory quantifiers
Source: PLoS One. 2020 Jul 27;15(7):e0229425. doi: 10.1371/journal.pone.0229425 (PMC7384625; doi:10.1371/journal.pone.0229425)
Supplement: S1 File — (PDF) [file pone.0229425.s008.pdf]

## Supporting information

**S1 Appendix. Details of the ACI and $H_a$ calculations.**

To obtain the ACI index of an audio segment, we first need to calculate the signal spectrogram using the STFT (Short-Time Fourier Transform) algorithm. The spectrogram is a data structure in a matrix format with $i$ rows and $t$ columns, where $i$ indicates the number of frequency bands. A key step of this index is to calculate the rate of change in each frequency band of the spectrogram ($|\Delta f^i|/\Delta t$). In other words, ACI is based on the first-order derivative of the frequency bands regarding time. Thus, each line of the spectrogram is summed up to generate a vector with $i$ values. The final ACI value is obtained by adding all these elements and normalizing by the total energy of the spectrogram, as follows:

$$ACI = \frac{\sum_{\forall i} \sum_{\forall t} |\Delta f_t^i|}{\sum_{\forall i} \sum_{\forall t} |f_t^i|}, \tag{1}$$

where $f^i$ denotes the i-th frequency band, $|\Delta f_t^i| = |f_{t+1}^i - f_t^i|$ is the difference of energy between two consecutive time stamps within the same frequency band, and $t$ are time stamps. The denominator of this fraction quantifies the total energy of the spectrogram.

The simple calculation of the ACI can be extended by averaging the segments of the spectrogram when a recording is too long. The steps are: segment the original spectrogram into smaller spectrograms, obtain the ACI for each segment, and finally, average all segments to obtain the $\overline{\text{ACI}}$.

The Acoustic Entropy Index assumes that the acoustic richness increases along with the diversity of animals singing in a given soundscape. Acoustic entropy $H_a$ is the product of two sub-indices: temporal entropy $H_t$ and spectral entropy $H_f$. These two indices are calculated using the following equations:

$$H_t = \frac{-1}{\log N} \sum_{t=1}^{N} A(t) \log A(t), \tag{2}$$

where $N$ is the temporal length of the signal, $A(t)$ is the probability mass function of the amplitude envelope obtained with the Hilbert Transform, and

$$H_f = \frac{-1}{\log(F/2)} \sum_{f=1}^{F/2} S(f) \log S(f), \tag{3}$$

where $F$ is the number of points used by the FFT algorithm to obtain the frequency spectrum $S(f)$. Here, the positive half of the signal's frequency spectrum is used as the probability mass function $S$.

**S2 Appendix. Efficient autocorrelation calculation.** The relation between the Power Spectral Density (PSD) of a signal and its autocorrelation function is described by the Wiener-Khintchine theorem [1] as:

$$R_{xx}(\tau) = \int_{-\infty}^{\infty} S_{xx}(f) e^{i2\pi f \tau} \mathrm{d}f \tag{4}$$

where $S_{xx}(f)$ is the PSD. Therefore, the autocorrelation coefficients expressed by Eq. 1 can be efficiently calculated using the Fast Fourier Transform algorithm (FFT) applying the three following steps:

1. apply the FFT on $X(t)$ to obtain its frequency spectrum, $F(f) = \text{FFT}[X(t)]$;
2. multiply $F(f)$ by its complex conjugate to get the PSD, $S_{xx}(f) = F(f)F^*(f)$; and finally
3. obtain $R_{xx}(\tau)$ using the Inverse FFT, $R(\tau) = \text{IFFT}[S(f)]$.

This procedure allows computing the autocorrelation from the raw data $X(t)$ with two FFT's. It is worth noting that, a naive procedure using Eq. 1 has a computational complexity order $O(n^2)$, whereas using the FFT has complexity $O(n \log_2 n)$. Its reduced cost makes this procedure attractive to be embedded into small sensor motes. Moreover, the minimum computational complexity is reached when the length of $R_{xx}(\tau)$ is a power of 2, justifying why we chose the values 32, 64, 128, 256 and 512 in our experiments.

**S3 Appendix. Normalized Jensen-Shannon divergence.** The quantity expressed by Eq. 5, known as "disequilibrium", can be rewritten as:

$$Q[P, P_e] = \frac{J[P, P_e]}{Q_{max}}, \tag{5}$$

where $Q_0 = {}^1/_{Q_{max}}$, then

$$Q_0 = -2 \left\{ \frac{\tau_{max} + 1}{\tau_{max}} \log(\tau_{max} + 1) - 2\log(2\tau_{max}) + \log(\tau_{max}) \right\}^{-1} \tag{6}$$

is the maximum possible value of $Q[P, P_e]$, obtained when only one component of $P$ is equal to one, and all the others are equal to zero [2]. This means $\lambda_1 = 1$ and $\lambda_{2:\tau_{max}} = 0$ in our definition.

**S4 Appendix. Correlated stochastic noises**

Colored noises, also known as correlated stochastic noises, are present in almost every acoustic signal recorded in a rainforest environment [3, 4]. This class of noise typically represents natural phenomena. The following $\alpha$ values determine some common types of noise:

1. $\alpha = 0$ models the white noise containing an equal amount of energy in all frequency bands;
2. $\alpha = 1$ models the pink noise with equal sound pressure levels in each octave band decreases in energy as the frequency increases; and
3. $\alpha = 2$ models the red (or brown) noise, which is common in oceanographic recordings, it describes the ambient underwater noise from distant sources

Values of $1 \leq \alpha \leq 2$ include several low-frequency natural phenomena recorded by the microphone, for example, rain or wind. Here, illustrating these noises becomes a reference curve to determine which ecoacoustic signal samples have characteristics of colored environmental noise.

# References


1. Ricker DW. Echo Signal Processing. The Springer International Series in Engineering and Computer Science. Springer US; 2003.

2. Ribeiro HV, Zunino L, Lenzi EK, Santoro PA, Mendes RS. Complexity-Entropy Causality Plane as a Complexity Measure for Two-Dimensional Patterns. PLOS ONE. 2012;7(8):1–9. doi:10.1371/journal.pone.0040689.

3. Colonna JG, Nakamura EF, Rosso OA. Feature evaluation for unsupervised bioacoustic signal segmentation of anuran calls. Expert Systems with Applications. 2018;106:107–120. doi:10.1016/j.eswa.2018.03.062.

4. Colonna JG, Nakamura EF. Unsupervised selection of the singular spectrum components based on information theory for bioacoustic signal filtering. Digital Signal Processing. 2018;82:64 – 79. doi:10.1016/j.dsp.2018.07.009.
