# Supplementary material for: Estimating ecoacoustic activity in the Amazon rainforest through Information Theory quantifiers
Source: PLoS One. 2020 Jul 27;15(7):e0229425. doi: 10.1371/journal.pone.0229425 (PMC7384625; doi:10.1371/journal.pone.0229425)
Supplement: Attachment — Submitted filename: Response to Reviewers.pdf [file pone.0229425.s009.pdf]

J. G. Colonna
Instituto de Computação (Icomp)
Universidade Federal do Amazonas (UFAM)
Av. Rodrigo Otavio 6200, CP 69067-005
Manaus (AM), Brazil
✉ *juancolonna@icomp.ufam.edu.br*

May 30, 2020

Dear Plos One members of the editorial board and reviewers,

We are writing this response letter concerning the submission ID PONE-D-20-02986 entitled "The Amazon rainforest soundscape characterized through Information Theory quantifiers". We would like to thank all reviewers for their valuable comments and suggestions. We took all comments very seriously and worked on the manuscript to reflect their suggestions. The following are the changes that were requested along with our responses.

In accordance with the reviewers' recommendations, we divided the contribution of our article into two main points: first, we propose the use of Statistical Complexity for ecoacoustic signals, for that we had to define and validate a new methodology for ecoacoustic signal processing, and, second, we carried out an analysis with real data from an ecological preservation area in the Amazon.

We would like to mention that we have no interest in promoting competition between existing authors or indexes. Our goal is to develop a new tool to analyze ecoacoustic data using a well-established concept from Physics. We believe that our contribution is complementary to the existing indices and helps to show a different perspective.

Reviewer #1:

- The title of the paper could be more informative.
  Thanks for this suggestion, we propose a new title.
- Very interesting paper, but are the authors aware that the name of the index is too close to ACI index? This could create confusion, not competition. I suggest to change the name of the index to better differentiate it for the future literature (when possible).
  To avoid confusion we are proposing a new name to our index, now it is called Ecoacoustic Global Complexity Index. We decided to add the key term "Global" since we are using global information quantifiers.
- The result section is too long and is very hard to follow it. I suggest to reshape and shorten when possible. The reader has difficulties to follow it in some parts.
  Thanks for the suggestion, we know that the article is long and the subject is not trivial to understand, so we did our best to improve the understanding, summarizing when possible, and we also did a overall review in the English writing.
- In the discussion a better and more extended comparison with the other two indices would be welcome.
  In this new version of our manuscript, we implemented the algorithms to calculate AEI and ACI. We also made comparisons using the reference samples and added a new appendix showing results using data from Mamirauá. The comparative results are presented mainly in the "Characterization of the reference samples" section. We also comment on these results in the discussion section.
- Line 30: I recommend to add an intermediate sentence explaining the necessity to propose a new index.
  We added a better contextualization related to the advantage of using Statistical Complexity in the introduction. Besides, the proposed index captures different acoustic dynamics that the other indexes do not. To see some examples, check figures 2, 4, 5, 8, 9, and the new figure Fig3.

- Line 32 to 64: It seems to me more a conclusion or an extended summary and not an introduction. I suggest to split in the methods and in part in the concluding comments. The content is ok but it seems to me that does not belong to an introduction. For instance, at line 32 “The proposed Ecoacoustic Complexity Index...” But this is the first time that the index is cited.
  Thanks for the observation. Indeed, some parts of the introduction sounds like a conclusion, so we decided to reorganize that section and some parts of the original text were relocated to the conclusions. The related work section was also updated to give the reader a general idea of the problem and its context. We also rewrote the Discussion section.
- Line 64: http://bit.ly/2m12PWc seems not to work.
  Our apologies about this issue, we used a tool to shorten the link and it caused problems. We updated the link.
- Line 101-104: The author say things that are not correct. For instance at line 103 there are no indices that can work after a hardware malfunction!
  It was our mistake, we rewrote this paragraph. The sentence was too strong, indeed. What we wanted to communicate was the higher degree of resilience of our index to glitches in the hardware, that would cause gaps in the audio recordings. But, for that it was enough to mention that our index is less sensitive to variations in the temporal length of the recordings, so we changed the statement to better communicate this characteristic.
- Line 117: Not to defend ACI but ACI produces an acoustic signature really different for white noise or heavy rains, or dawn chorus. Second, is absolutely wrong to say that ACI depends on the length of the recorded files, if you average the total ACI.
  Your comment was very important, as our intention was not to underestimate the ACI. We simply wanted to point out how it works. In an effort to be clearer, we added a new appendix with the equation that can help in understanding our remark about ACI. Furthermore, after reviewing the original article again, we also checked the Soundecology package in the R-cran language, and we implemented our version of this index. Now, suppose you have two signals of the same environment, in the same moment, one with 10 seconds and one with 60 seconds. In this case, even with similar spectral content, the absolute values of the ACI will be very different, laying on different scales, for the total sum of the derivative will add a different amount of information. After applying the normalization coefficient, the result will be place in an yet another different scale.
  On the other hand, as the reviewer pointed out, if we divide the recordings into small segments of time, all with the same duration, we will have a convergence to an average value. However, events with low frequency of occurrence will be neglected, and the average value will be skewed and flattened. Even considering the application of ACI to different samples length with this workaround, it seems to us that the result will not be as precise as it would be if ACI is applied to audio samples with a fixed length.
  Furthermore, considering the scenario of automatic computation of the index, by embedding it in a sensor node, EGCI does not demand preparation of the signal.
  As a conclusion, the comment was very important, for to answer it we enriched the paper with a solid comparison between EGCI and ACI.
- Line 121-122: Please modify to be consistent with the previous comments.
  We added a more complete explanation of ACI in the Related Works section, added new numerical comparisons that show how this point is distorted, and also added an appendix that shows its calculation.
- Line 412: This is not a new result, but is confirmed by ACI.
  Thanks for the comment, as it gave us the opportunity to clarify in the paper a semantic aspect of both indexes names. Although both indexes have "complexity" in their names, the ACI equations do not represent a formal calculation of complexity. In other words, complexity in ACI seems to refers to a qualifier, implied by the result of the index. While, for EGCI complexity is a quantifier well defined by the Information Theory. In this case, complexity as in EGCI benefits from the legacy of this area of Mathematics and Statistics.

We conclude, then, that the statistical complexity represented in the HxC plane correlates with the diversity in the recordings.
Again, thanks for the review, for we had the opportunity to add a sentence to clarify this point. To better understand the difference between ACI and EGCI, please refer to the new figures 3 and 13.

- Line 415: Also this is common to other well established metrics, where is the novelty? Please explain better
  Thanks a lot to ask, as it seems the text was not clear. The goal of the paragraph mentioned was to explicitly discuss the behavior of the EGCI, and to point out that the hypothesis raised about it has been validated.
  Although the main focus of the work was not a comparison or competition between acoustic indices - EGCI was intended to be complementary, by capturing new information - in this new version we did some comparisons.
  The main goal was to propose a signal processing methodology well suited for the HxC complexity plane from ecoacoustic recordings. None of the previous works did that, and by doing so EGCI is giving an objective meaning to the label complexity for those recordings.
  The second main contribution of the article is the analysis of actual environmental data.
  Your comment was very important, as we make the contributions of our work more explicit by rewriting the Introduction.
- Line 416: Please explain the position of the insects
  The effect of the insects was better commented on the results and experiment sections. We hope now the role of insects is more clear in the paper.
- Line 418-423. I am sorry, but this section is really confused and contradictory please rephrase
  We rewrote this sentence. For a better understanding, note that in the results sections we mentioned that insects increase entropy and can decrease complexity.
- Line 424: Again this is not a peculiarity of ECI but other indices have found these patterns.
  Maybe we were not clear enough. Although the main point here is not the comparison between indexes, but to discuss the interpretations of the proposed methodology, we would like to remark that EGCI has better adherence to the monitored soundscape.
  But, of course, you are right, as all valid indexes represent patterns (or they would not be valid indexes). But, we expect that the index is sensitive enough to uniquely map different natural phenomena to a specific pattern.
  To better clarify our point, we would like to note that, in the new figure 13, the regularity of the acoustic patterns shown by the ACI does not correspond to the observations in the field.
- Line 404: In this discussion it seems a confusion between what the ECI is able to marks and the character of the study areas. For clarity the authors should separate in the discussion these two parts that now are stirred.
  Thanks for the suggestion. In this new version we have improved this separation. However, it is worth noting that the main differences are explained while presenting the results.
- Line 448: I don't believe that this is an important point
  We respect you position, but let us try to better explain the importance of this point. There are some places in the world where the ecosystem changes radically, and at the same time, regularly, during the span of an year. Mamirauá is one of such places. During the flood season the whole area is covered by 6m of water, and during the dry season there are aplenty of dry land available. It is an average of 10 meters of difference in the water level. This means that species and environmental phenomena change dramatically, so the soundscape changes in accordance. The index captured this dynamic, being, for instance, capable to characterize, in the long run, how is the status of the ecosystem.
- Line 469-471: Looks not necessary, your paper has methodological characters and references to other biomes in Brazil contaminates the "universal" valence of the ECI.
  Thanks for this comment. We rewrite it as follows: "In future investigations, we would like to expand our data analysis to characterize the acoustic dynamics of other regions."

Reviewer #2:

- In this manuscript the authors describe a new ecoacoustic index and demonstrate how linking entropy and complexity over space and time adds value to our understanding of a soundscape. The impact of the work could be enhanced with better framing in the introduction (see below) and a clearer contrast between the ECI and other acoustic indices. Addressing these would help readers who are users of acoustic indices see the value of the contribution.
  Thanks for the suggestion. In this new version of our manuscript, we rewrote the introduction to make our contribution more explicit. We also added comparisons with other indices in the "Characterization of reference samples" section and the new appendices.
- Line 22 - There are far more than two ways to monitor change in animal populations! Visual surveys, camera traps, genetics sampling... I see where the authors are going, and it is a good direction, but the opening paragraph needs to be more inclusive of the broader research (at least as currently phrased).
  We rewrote that sentence. A broader context has been included in the Related works section.
- Line 72 - I would clarify the differences between the supervised and unsupervised methods. In particular, what the focus of each tool is in the field. Is it to describe the biological community or change in a population or is it to described the soundscape and how it might change? These are two complementary but different goals.
  Thanks for the clarification. The reviewer's comment is correct, supervised approaches are often used to track the status of some species, while unsupervised approaches quantify abundance through the diversity of patterns in the data. Although they are different approaches, there is a link between them. For example, changes in biological communities also change the soundscape; therefore, if the species monitored in a supervised manner do not manifest within a period, there will be changes in the soundscape. In our approach, we do not attempt to estimate the population. The focus is to discriminate the acoustic richness through the manifestation of regular and irregular patterns that are repeated in the recordings. Our initial comment on this subject was broad, just to contextualize the reader and to mention the different possibilities. However, we added clarifications in the introductory text.
- Line 105 - how are you defining acoustic richness? Does it compare to species richness in your application? Indeed clarity in what biological vs acoustic measures of focus and the relationship between them should be addressed.
  The acoustic richness is defined by the regularity (or irregularity) of the acoustic patterns in the recordings. This is the traditional way in which most related work defines the problem. There is a correlation between the diversity of species with different calls and acoustic diversity. This point can be seen in Figures 5, 6, and 8, which show that the presence or absence of so-called different species directly affects the acoustic complexity index. For example, figure 8 shows specifically the displacement of the index with the seasons and we know that between the flood and drought seasons there are different species.
- Line 228 - but the data from Saeur is said about to have the highest richness a s19 and s18. Is ecoacustic richness different then biodiversity richness? How are these applicable as measures of animal populations overtime (from the opening paragraph)?
  As pointed out by Sueur et. al. some recordings have greater entropy quantified by H_a. A value similar to H_a is obtained using our Von Neuman definition of entropy. To clarify this point, we add the new figure 3. Although s_{19} and s_{18} have high entropy, the complexity is not maximum, and, as explained in the related work section, this may be due to the increase in the uncorrelated noise or the spread of energy between the frequency bands. This is the main point that justifies complementing the analyzes using our measure of Statistical Complexity, recalling that increasing the diversity of regular acoustic patterns increases complexity and more patterns mean high abundance. In this sense, the main disadvantage are the irregular patterns that appear only once, which could increase entropy and slightly reduce complexity.
- Line 267 - this seems like the most useful interpretation. Perhaps in fig 1 use this term, complexity plane, and describe and interpret it for readers coming from the use/application side to see how it adds value to other measures.
  Thank you for noticing this. Our main motivation was to bring the concepts of complexity

closer to the community interested in ecoacoustics. To bring the benefits, it was necessary to define the correct methodology to obtain the PMF from the recordings.

- Perhaps also show how the ECI compares to the indexes mentioned above (and others noted below) to show how the additional information from the ECI adds value.
  The ECI is the composition of three quantifiers: Entropy, Divergence, and Complexity. Each of these quantifiers adds value to the data analysis and highlights different aspects of the recordings and the soundscape. In addition to the new numerical comparisons added, we also complement the Related Work section by adding the suggested references: the NDSI (Kasten et al. 2012), the BAI (Boelman et al. 2007), the ADI and AEI (Villanueva-Rivera et al. 2011).

The following comments were related to language inconsistencies, grammatical errors, typos, formatting problems, or related to the above answers. Therefore, we group these comments. All of them have been revised and corrected.

Line 2: Environmental better than ecological?

Line 6: soundscape's biodiversity seems to me not appropriate terminology.

Line 17: species richness,..., and insects? Seems to me not well linked.

Line 28: not only diversity but also dynamics, daily and seasonal cycles, etc. please rephrase.

Line 38: frequency better than spectral?

Line 78: please add some references

Line 104, the index has been proposed to evaluate the level of complexity inside a soundscape, or if you prefer it measures the amount of acoustic information present at every frequency bin.

Line 109: should assumed? Or should assume.

Line 410: I don't understand, please rephrase.

Line 462: the http does not work?

Line 468: ecoacoustics landscape? Why not soundscape?

Line 19 - The first two sentences are both true, but not logically linked. The recent paper (citation #1) only came out last year, thus the observations have not driven the behavior in part two. A simple solution would be to flip these, with the second a narrower application of monitoring animal populations. ** however after reading the whole paper, this seems not to be the focus.

In summary, we rewrote a substantial part of our manuscript, addressing all the mentioned issues. We did our best to bring to the reader a more fluid text. We kindly request a second review. We do believe that we managed to provide all the necessary improvements and information requested.

In case of any doubt, please feel free to contact us anytime.

Sincerely,

Juan G. Colonna (corresponding author),
J. Reginaldo H. Carvalho, and
Osvaldo A. Rosso
